# The impact of functional independence, well‐being, and support systems on caregiver burden and self‐efficacy in dementia care

**DOI:** 10.1002/alz70858_106546

**Published:** 2025-12-26

**Authors:** Raluca Sfetcu, Wolfgang Kratky, Julia Himmelsbach, Francesca Gris, Ikse Bierhoff, Catalina Tudose, Serena Cancellieri, Judith Goldgruber, Markus Garschall, Demicare group

**Affiliations:** ^1^ GBHI TCD, Dublin, Dublin, Ireland; ^2^ Geriatric Health Care Centers Graz, Graz, Styria, Austria; ^3^ AIT Austrian Institute of Technology GmbH, Vienna, Austria; ^4^ INRCA, Ancona, Italy, Italy; ^5^ VIALNS, Utrecht, Netherlands, Netherlands; ^6^ Carol Davila University of Medicine and Pharmacy, Bucharest, Romania; ^7^ GGZ Graz, Graz, Austria, Austria; ^8^ AIT Austrian Institute of Technology GmbH, Vienna, Austria, Austria

## Abstract

**Background:**

Caring for a person with dementia presents significant physical, emotional, and psychological challenges. Understanding the interplay between the functional independence of the person with dementia and caregiver‐related factors such as burden, well‐being, and self‐efficacy is crucial for developing effective support interventions. This study explores key predictors of caregiver burden and efficacy using baseline data from a mixed experimental‐control cohort.

**Method:**

A secondary data analysis was conducted using baseline measures from a dementia caregiving study. Correlation and regression analyses were performed to examine the relationships between the functional independence of the person with dementia and caregiver‐related factors, including well‐being, burden, and self‐efficacy. Additional variables, including service use and social support, were assessed for their contribution to caregiving outcomes.

**Results:**

Caregiver burden was strongly associated with caregiver well‐being, indicating that caregivers with lower mental well‐being experience higher perceived burden. The functional independence of the person with dementia alone was not a strong predictor of caregiver burden, suggesting that caregiver distress is influenced more by psychological and social factors than by the care recipient's physical function. Caregiving efficacy was moderately associated with well‐being and negatively linked to burden, suggesting that caregivers with greater well‐being feel more confident in their caregiving role. Additional factors, including service use, social support, and caregiving frequency, contributed to explaining variations in caregiver burden, highlighting the importance of multi‐dimensional support.

**Conclusion:**

Caregiver burden is not solely determined by the functional status of the person with dementia but is strongly linked to caregiver psychological well‐being, support systems, and perceived efficacy. Interventions targeting caregiver mental health, structured support networks, and access to resources may be key in reducing burden and improving caregiving confidence. Future research should explore longitudinal trends and intervention‐based strategies to enhance caregiver resilience.

